# Associations Between Stevens–Johnson Syndrome and Infection: Overview of Pharmacoepidemiological Studies

**DOI:** 10.3389/fmed.2021.644871

**Published:** 2021-03-26

**Authors:** Takuya Imatoh, Yoshiro Saito

**Affiliations:** ^1^Division of Cohort Research, Center for Public Health Sciences, National Cancer Center, Tokyo, Japan; ^2^Division of Medicinal Safety Science, National Institute of Health Sciences, Kanagawa, Japan

**Keywords:** Stevens–Jonhson syndrome, toxic epidermal necrolysis, infection, pharmacoepidaemiology, real world evidence

## Abstract

Stevens–Johnson syndrome and toxic epidermal necrolysis (SJS/TEN) are classified as type B adverse drug reactions, and are severe, potentially fatal rare disorders. However, the pathogenesis of SJS/TEN is not fully understood. The onset of SJS/TEN is triggered by the immune system in response to antigens with or by drugs. As activation of the immune system is important, infection could be a risk factor for the onset of SJS/TEN. Based on the hypothesis that infections induce the onset of SJS/TEN, we conducted pharmacoepidemiological investigations using two spontaneous adverse drug reaction reporting databases (Japanese Adverse Drug Event Report database and Food and Drug Administration Adverse Event Reporting System) and Japanese medical information database. These data suggest that infection could be a risk factor for the development of SJS/TEN. In this mini-review, we discuss the association between infection and the development of SJS/TEN.

## Introduction

Stevens–Johnson syndrome (SJS) and the related disease toxic epidermal necrolysis (TEN) are two of the most serious adverse reactions caused by drugs. SJS is characterized by high fever, malaise, and a rapidly developing, blistering exanthema of macular papules and target-like lesions, accompanied by mucosal involvement. According to the World Allergy Organizations (WAO) definition of 2014, SJS/TEN are defined by the degree of skin detachment: SJS/TEN are defined as skin involvement of <10 and >30%, respectively, and that of SJS/TEN overlap as 10–30% skin involvement.

SJS/TEN are known as idiosyncratic drug reactions. They are rare and unpredictable. Therefore, serious idiosyncratic drug reaction is one of the cause of withdrawal of drug from the market. SJS/TEN are rare but life-threatening severe cutaneous adverse reactions, which are mainly induced by a variety of drugs. SJS/TEN occur in all ages, races, and sexes (1), with incidences ranging from 0.4 to 1.2 and 1.2 to 6 per million person-years. A USA-based study analyzing nationwide inpatient records from 2009 to 2012 calculated an incidence per million inhabitants of 8.61–9.69 for SJS, 1.46–1.84 for SJS/TEN overlap, and 1.58–2.26 for TEN (2). Epidemiological studies have calculated a TEN incidence rate of 1.2 per 1 million inhabitants per year for France (3) and of 0.93 per 1 million inhabitants per year for Germany (4). However, Frey et al. recently estimated that Asian patients were at a two-fold higher risk of SJS/TEN when compared with Caucasian patients (5). The incidence of SJS ranged from 3.3–4.1 per million people per year in Taiwan between 2000 and 2008 (6). Approximately 800–1,000 cases of SJS and 500–700 cases of TEN are reported annually in Japan (7).

Some drugs have been associated with the onset of SJS/TEN. Phenytoin, carbamazepine, and phenobarbital have been reported as common causes of SJS in a previous case control study (8). Additionally, sulfamethoxazol/trimethoprim, sulfonamides (sulfasalazine, sulfadiazine, sulfadoxine, and sulfafurazole) and oxicam-type non-steroidal anti-inflammatory drugs (NSAIDs) (meloxicam, piroxicam, and tenoxicam) are known to have a high risk of inducing SJS/TEN. A large proportion of suspected drug is comprised cold medicines. In particular, SJS/TEN with severe ocular complications (SOC) associated with cold medicine were reported in several studies (9, 10). In addition to conventional drugs, herbal remedies and new biologicals have been considered as causative agents. Recently, lamotrigine and zonisamide have been reported as major causative drugs of SJS/TEN (7).

The pathogenesis of SJS/TEN is not yet fully understood, but it is thought to be immune-mediated. The role of the T-cell-mediated immune response in the pathogenesis of SJS/TEN has been firmly established. In particular, the human leukocyte antigen (HLA) system plays an important role in the pathogenesis of SJS/TEN, as some drugs may bind directly to the HLA-complex and cause self-reactivity due to the drug-modified HLA-peptide repertoire (11). There are numerous reported associations between some *HLA* alleles and SJS/TEN (12–14).

In this mini-review, we have focused to analyze on infections, that is, the role of the immune response to viruses and bacteria in SJS/TEN. Previous studies have reported associations between some infections and the onset of SJS/TEN. However, most were clinical studies and case reports (15–19) with very few pharmacoepidemiological studies. Therefore, we conducted a pharmacoepidemiological study to elucidate the association between infection and SJS/TEN using the Japanese Adverse Drug Event Report (JADER) database. We also used the Food and Drug Administration (FDA) Adverse Events Reporting System (FAERS) database to compare these associations in the US, Europe, Japan, and other countries. Based on these findings, we discuss the association between infection and the development of SJS/TEN.

### Results of a Pharmacoepidemiological Study Using the JADER Database

First, to elucidate the association between infection and SJS/TEN, we conducted a pharmacoepidemiological study using the JADER database (20).

JADER is a large published spontaneous reporting database for drug adverse reactions that was established by Japan's Pharmaceuticals and Medical Device Agency (PMDA) for pharmacovigilance activities. The dataset can be accessed directly on: http://www.info.pmda.go.jp/fukusayoudb/CsvDownload.jsp (available in Japanese only), and compose of four relational tables: DEMO, DRUG, REAC, and HIST. The tables contain the following information: demographic information of patients including, sex and age (DEMO table), drug information used in patients (DRUG table), adverse drug information that have occurred in patients (REAC table) and primary disease information (HIST table). Each table was connected using the ID number of each recorded case.

We used the November 2014 version of JADER and extracted the records of 177,659 cases of ADRs reported from 2009–2013. The reported cases were classified into three categories (anti-infectious drug group, concomitant infection group, and non-infection group) based on the presence of anti-infectious drugs (either as primary suspected drug or concomitant drug) and infectious disease. We defined the cases whose clinical outcome was “death,” “unrecovered,” or “with sequela” as severe. We assessed the association between SJS/TEN and the presence or seriousness of infection using logistic regression analysis. Logistic regression analysis showed a significant positive association between the infection status and onset of SJS/TEN compared to the non-infection group. Odds ratios (OR) were 2.04 [95% confidence interval (CI) (1.85–2.24)] for the anti-infectious drug group and 2.44 [95% CI (2.21–2.69)] for the concomitant infection group, respectively. Moreover, a significantly positive association between infection and SJS/TEN severity was observed [OR 1.48; 95% CI (1.10–1.98)]. These results suggest that SJS/TEN is strongly associated with infection in this Japanese dataset.

### Results of a Pharmacoepidemiological Study Using a Japanese Medical Information Database

Spontaneous drug adverse reaction databases have considerable biases. Limited information is available in standardized spontaneous reports (21) and only a fraction of adverse drug events are identified and reported (22). There is a growing need for real-world data in medical and healthcare research. Big data in healthcare may be comprised of massive amounts of information from various sources, including electronic health records (EHRs), administrative or claims data, and data from self-monitoring devices. The utilization of electronic medical record databases for drug safety assessment has been extensively discussed (23–25). In Japan, a new medical information database network (designated MID-NET®) to provide real-world data for drug safety assessments was officially launched in April 2018 (26). This network was designed and developed by the Ministry of Health, Labor and Welfare, and the PMDA in collaboration with 23 hospitals of 10 healthcare organizations across Japan. Thus, real-world data is an important source of information for the detection of adverse drug reactions.

We used a medical information database in Japan to confirm the association between infection and the onset of SJS/TEN (27). We used commercial medical information data collected by Medical Data Vision (MDV Co., Ltd). The medical information database covers more than 30 million patients in more than 400 Japanese hospitals and includes health claims, pharmacy claims and the diagnosis procedure combination (DPC) data.

Since antipyretic analgesics, including acetaminophen and loxoprofen sodium, are widely prescribed and are known as major suspected drugs for SJS/TEN (28), we conducted a matched nested case-control study to elucidate the association between concurrent infection and the onset of SJS/TEN in patients prescribed antipyretic analgesics. We used the data of 4,112,055 patients prescribing antipyretic analgesics between January 2014 and December 2015. The presence of International Classification of Diseases, 10th revision (ICD-10) codes for SJS/TEN (L511, L512) was defined as SJS/TEN. 553 (0.01%) were diagnosed with SJS/TEN. Among them, 131 patients who had been prescribed antipyretic analgesics during the month prior to the defined date of SJS/TEN onset were identified. Moreover, to minimize the impact of antibiotics agents, we excluded the patients who had been prescribed top 10 suspected antibiotics and viral and fungal agents (levofloxacin, clarithromycin, amoxicillin, galenoxacin, sefkapen, sulfamethoxazole/trimethoprim, azithromycin, ceftriaxone, cefditoren and vancomycin) during the month prior to the index date.

In a matched nested case-control study, for each case, three controls were randomly matched with the case for age at index date (onset of SJS/TEN) and sex. Infection was defined the diagnosis of infection (bacterial, viral, and fungal infection) during the month prior to the index date, which co-existed with one or more prescription claims for antibiotic agents (antibiotics-bacterial, virus, and fungal infection agents) in the same month. We calculated odds ratios and 95% CIs using conditional logistic regression to estimate the association between SJS/TEN and infection on patients prescribing antipyretic analgesics.

According to conditional logistic regression analysis, patients with infection and prescribing antipyretic analgesics had significantly increased the risk of onset of SJS/TEN [adjusted OR 5.59, 95% CI (2.01–15.51)] (Table 1). Similar to the results of our study using the JADER, It suggests that infection may increase the risk for the onset of SJS/TEN in patients taking antipyretic analgesics.

**Table 1:** Odds ratios for the combination between antipyretic analgesics and infection in matched nested case control analysis of SJS/TEN.

			**Cases**		**Controls**		**Crude**		Adjusted^*^	
			***n***	**%**	***n***	**%**	**OR**	**95% CI**	**OR**	**95% CI**
Antipyretic analgesics/Infection	−	−	42	32.1	183	46.6	1.00	(Reference)	1.00	(Reference)
	−	+	18	13.7	58	14.8	1.61	(1.03–2.52)	1.13	(0.43–2.95)
	+	−	41	31.3	119	30.3	1.76	(0.75–4.11)	1.61	(1.00–2.61)
	+	+	30	22.9	33	8.4	7.42	(3.10–17.79)	5.59	(2.01–15.51)
CCI	Low (0–1)		77	58.8	294	74.8	1.00	(Reference)	1.00	(Reference)
	Medium (1–3)		37	28.2	68	17.3	2.22	(1.35–3.67)	1.55	(0.88–2.75)
	High (3+)		17	13.0	31	7.9	2.17	(1.14–4.13)	1.14	(0.42–2.49)
Corticosteroid use	No		65	49.6	308	78.4	1.00	(Reference)	1.00	(Reference)
	Yes		65	49.6	60	15.3	6.30	(3.80–10.44)	5.46	(3.20–9.32)

*OR, odds ratio; 95% CI, 95% confidence interval; CCI, Charlson comorbidity index*.

* *Adjusted for Charlson comorbidity index and corticosteroid use*.

*Reprinted with permission from (25). Copyright (2020), The American College of Clinical Pharmacology*.

### Results of a Pharmacoepidemiological Study Using FAERS

Based on both JADER and Japanese medical information databases, an association was found between infection and the onset of SJS/TEN. We next conducted a pharmacoepidemiological study using FAERS to elucidate the association between infection and the onset of SJS/TEN in other countries.

The FAERS database contains >14 million adverse event reports, medication error reports and product quality complaints resulting in adverse events that were submitted to the FDA in the USA and other countries and has increased to over 1.8 million reports per year. Case report information is separated into seven tables, which contain details on patient demographics and administrative information (DEMO), medication and biological products used (DRUG), adverse drug reactions (REAC), patient outcomes (OUTC), report sources (RPSR), drug therapy start and end dates (THER), and indication (INDI). The database is made publicly available as a quarterly download on the FDA website.

We obtained data from case reports received by the FDA between January 1, 2007 and June 30, 2017. Duplicate reports were removed, and drug names were modified based on modified World Health Organization (WHO) drug dictionaries. To identify infections, we used the DRUG and INDI files, which include two drug variables and an indications variable, defined as follows: (1) DRUG_CODE: code of medicinal product; (2) ROLE_COD: code for drug's reported role in event, and (3) INDI_PT: PT code of indications. We categorized infection into three categories, defined as follows: (1) primary suspected anti-infectious drug group, (2) concomitant infection group, and (3) non-infection group. We identified individual SJS/TEN based on the MedDRA (version 20.1) preferred terms in the FAERS database. The ORs and 95% CIs were calculated using logistic regression analysis.

We observed significant associations between infections and SJS/TEN in the US and England (Table 2). In particular, a strong association was observed in the US. [US: OR for primary suspected anti-infectious drug group 8.21, 95% CI (7.60–8.87), OR for concomitant infection group 3.69, 95% CI (3.32–4.10); England: OR for primary suspected anti-infectious drug group 3.30, 95% CI (2.82–3.87), OR for concomitant infection group 3.69, 95% CI (2.82–3.98)]. However, there was no association between infection and SJS/TEN in China. [OR for primary suspected anti-infectious drug group 0.89, 95% CI (0.53–1.48), OR for concomitant infection group 1.13, 95% CI (0.59–2.18)].

**Table 2:** Odds ratios for the association between infection and SJS/TEN in the FAERS database.

		**Cases**	**Non-cases**	**Odds ratio**	**(95% CI)**
Japan	Anti-infectious drug	403	27,513	2.05	(1.81–2.33)
	Concomitant infection	361	23,517	2.04	(1.80–2.32)
	Non-infection	1,146	177,082	1.00	(Reference)
The United States	Anti-infectious drug	1,302	203,782	8.21	(7.60–8.87)
	Concomitant infection	526	182,813	3.69	(3.32–4.10)
	Non-infection	4,134	4,617,902	1.00	(Reference)
England	Anti-infectious drug	268	28,227	3.30	(2.82–3.87)
	Concomitant infection	215	21,021	3.35	(2.82–3.98)
	Non-infection	599	230,352	1.00	(Reference)
China	Anti-infectious drug	21	5,125	0.89	(0.53–1.48)
	Concomitant infection	13	2,019	1.13	(0.59–2.18)
	Non-infection	121	30,864	1.00	(Reference)
All countries (except Japan)	Anti-infectious drug	3,430	383,970	6.92	(6.59–7.28)
	Concomitant infection	2,129	318,665	4.73	(4.66–5.24)
	Non-infection	8,832	6,535,291	1.00	(Reference)

*Adjusted for age (by 20-year-old class) and sex*.

*95% CI, 95% confidence interval*.

## Discussion

We consulted three medical databases JADER, a medical information database in Japan, and FAERS, to analyze the association between infection and SJS/TEN. Based on the available information, there appears to be a strong statistical association between infection and the onset of SJS/TEN, in Western countries, as well as in Japan.

Cutaneous diseases, including drug reactions, are increased up to 100-fold in persons with human immunodeficiency virus (HIV) (29). Infectious agents, such as a variant of Coxsackie virus A6 and *Mycoplasma pneumoniae*, have been previously reported to cause SJS/TEN (16, 30, 31). Furthermore, 56% of SJS patients had an antecedent upper respiratory tract infection or non-specific viral infection (19, 32). Recently, a study using microbiological analysis reported that a higher proportion of pathogenic microorganisms, including *Pseudomonas* spp., *Staphylococcus* spp., *Streptococcus* spp., and *Acinetobacter* spp., was observed in the SJS patient group (33). Meanwhile, several pharmacoepidemiological studies have reported that some classes of antibiotics are associated with the occurrence of SJS/TEN (3, 5, 34). Previous studies that used EHRs have reported that more than half of the SJS/TEN cases are attributed to antibiotics (35).

We found that the association between infection and SJS/TEN differed between countries. The frequency of HLA alleles differs among ethnicities. For example, HLA-B^*^1502 is common in East Asians (6.9%) (36), and the FDA recommends screening this allele in individuals of Asian ancestry before initiating treatment with carbamazepine.

SJS/TEN is thought to be a type IV hypersensitivity reaction, in which a drug or its metabolite stimulates cytotoxic T-cells and helper T cells. The specific molecular mechanisms driving this reaction have only been elucidated in a handful of cases, and the pathogenesis of SJS/TEN is not completely clear. Numerous studies have reported that HLA and T-cell receptors play an important role in the immune mechanisms of SJS/TEN. Several concepts have been proposed to explain the pathogenesis of severe cutaneous adverse drug reactions. In the hapten concept, small molecules called haptens elicit an immune response only when attached to proteins. The “p-i” concept postulates that the suspected drugs can stimulate cells by binding directly and reversibly to immune receptors. The altered peptide repertoire model suggests that drugs alter the antigen by binding to the human leukocyte antigen pocket. Recently, White et al. proposed that some T-cell mediated hypersensitivity reactions likely represent another example of heterologous immunity (37). Another possibility is that innate immunity-related molecules such as toll-like receptors might be activated by the infections and their involvement of specific immune reactions to the drug for SJS/TEN onset. The pathogenesis of SJS/TEN clearly requires further investigation.

There are many case reports and small sample-size clinical studies regarding the association between infection and SJS/TEN. Epidemiological studies with larger sample sizes are extremely rare. Although large-scale epidemiological studies have been performed in the United States, Europe, and Korea (5, 38, 39), little has been reported on the association between infection and SJS/TEN. We conducted large-scale epidemiological studies using Japanese medical data from spontaneous reporting adverse databases and medical information databases. While JADER or FAERS are useful to investigate the risk factors for adverse drug reactions, as the incidences of SJS and TEN are very rare, these databases have some inherent limitations. These limitations include underreporting, biased reporting rates, incomplete patient information, and indeterminate population exposure, and create the need for complementary data sources and methods (40). There are two major limitations that are unique to the FAERS database. First, the drug nomenclature consists of medication brands and trade names, generic or non-proprietary names, and chemical or scientific names with frequent misspellings. Second, there are a lot of duplicates and multiple follow-up reports (41). Consequently, the interpretation of these findings requires careful consideration.

We also conducted an epidemiological study using a Japanese medical information database. The limitations of real-world evidence studies can include low internal validity, lack of quality control surrounding data collection, and susceptibility to multiple sources of bias for comparing outcomes. As these database were collected retrospectively, there may be some potential confounding factors that were not analyzed. In our medical information database study, even though the patients prescribing top 10 suspected antibiotics and viral and fungal agents in SJS/TEN were excluded, the significant association remained. However, it was not possible to completely distinguish whether the associations were caused by the infection, or were a direct consequence of the antibiotic agents. Hence, additional evidence is needed to elucidate the association between infection and SJS/TEN.

In summary, we reviewed our pharmacoepidemiological studies on the association between infection and SJS/TEN. These studies indicated that there is a strong association between infection and the onset of SJS/TEN, in Japan and other countries analyzed. Overall, infection may be an important risk factor for SJS/TEN worldwide.

## Author Contributions

All authors listed have made a substantial, direct and intellectual contribution to the work, and approved it for publication.

## Conflict of Interest

The authors declare that the research was conducted in the absence of any commercial or financial relationships that could be construed as a potential conflict of interest.
